# Characterising the proteomic response of mushroom pathogen *Lecanicillium fungicola* to *Bacillus velezensis* QST 713 and Kos biocontrol agents

**DOI:** 10.1007/s10658-022-02482-1

**Published:** 2022-04-22

**Authors:** Joy Clarke, Helen Grogan, David Fitzpatrick, Kevin Kavanagh

**Affiliations:** 1 Department of Biology, Maynooth University, Maynooth, Kildare Ireland; 2 Teagasc, Horticulture Development Department, Ashtown Research Centre, Dublin 15, Ireland

**Keywords:** *Agaricus bisporus*, Biocontrol, *Lecanicillium fungicola*, *Bacillus velezensis*, Proteomics, Dry bubble disease

## Abstract

The fungal pathogen *Lecanicillium fungicola* causes dry bubble disease in *Agaricus bisporus* cultivation and affected mushrooms significantly reduce the yield and revenue for mushroom growers. Biocontrol agents may represent an alternative and more environmentally friendly treatment option to help control dry bubble on mushroom farms. Serenade ® is a commercially available biocontrol product used for disease treatment in plant crops. In this work, the in vitro response of *L. fungicola* to the bacterial strain active in Serenade, *Bacillus velezensis* (QST 713) and a newly isolated *B. velezensis* strain (Kos) was assessed. *B. velezensis* (QST713 and Kos) both produced zones of inhibition on plate cultures of *L. fungicola*, reduced the mycelium growth in liquid cultures and damaged the morphology and structure of *L. fungicola* hyphae. The proteomic response of the pathogen against these biocontrol strains was also investigated. Proteins involved in growth and translation such as 60S ribosomal protein L21-A (−32-fold) and 40S ribosomal protein S30 (−17-fold) were reduced in abundance in *B. velezensis* QST 713 treated samples, while proteins involved in a stress response were increased (norsolorinic acid reductase B (47-fold), isocitrate lyase (11-fold) and isovaleryl-CoA dehydrogenase (8-fold). *L. fungicola* was found to have a similar proteomic response when exposed to *B. velezensis* (Kos). This work provides information on the response of *L. fungicola* to *B. velezensis* (QST 713) and indicates the potential of *B. velezensis* Kos as a novel biocontrol agent.

**Supplementary Information:**

The online version contains supplementary material available at 10.1007/s10658-022-02482-1.

## Introduction

*Lecanicillium fungicola* (Preuss), Zare and Gams [synonyms: *Verticillium fungicola* (Preuss)*,* Hassebrauk] is a pathogenic fungus which causes dry bubble disease during white button mushroom cultivation (*Agaricus bisporus*) (Lange) Imbach. *A. bisporus* is one of five main commercially grown species, which together account for 85% of the world’s mushroom supply. *Lentinula* accounts for 22% of world mushroom production while *A. bisporus* currently contributes 15% (Royse et al., 2017). *L. fungicola* infection results in severely deformed crops which greatly reduce the yield of marketable mushrooms (Berendsen et al., 2010). An increase in fungicide resistant *L. fungicola* strains has meant that this pathogen is currently one of the biggest problems in commercial mushroom production. The application of biocontrol agents is being investigated as a potential alternative to fungicide use. Other pathogens which are responsible for mushroom disease and potentially may require biocontrol treatment in the future include *Trichoderma aggressivium* (Green Mould Disease), *Cladobotryum* species (Cobweb Disease) and *Mycogyne perniciosa* (Wet Bubble Disease) (Fletcher & Gaze, 2007; Largeteau & Savoie, 2010).

*L. fungicola* was previously referred to as *Verticillium fungicola* (Gams & Van Zaayen, 1982; Hassebrauk, 1936). In 2008, Zare and Gams confirmed that *V. fungicola* was more closely related to a plant pathogenic genus *Lecanicillium*, thus *Verticillium* was renamed to *Lecanicillium* (Zare & Gams, 2008). The variety *var. fungicola* is mostly associated with disease incidence in Europe, while *var. aleophilum* occurs mostly in the USA and Canada (Largeteau et al., 2004). While *L. fungicola* has been isolated from a number of other cultivated mushroom species (e.g. *Pleurotus ostreatus, Pleurotus sapidus, Coltricha perennis*) (Marlowe & Romaine, 1982), it is rarely identified growing on wild mushrooms (Berendsen et al., 2012).

Mushroom casing, which is a mixture of peat and a neutralising agent such as sugar-beet lime or ground limestone, is easily contaminated during preparation and therefore it is commonly considered as a primary source of infection of *L. fungicola* on mushroom farms (Berendsen et al., 2012; Carrasco et al., 2019; Cross & Jacobs, 1969). Centralised casing production has vastly improved in Europe and casing is less likely to be contaminated at source, however prepared casing is still open to contamination. Possible primary sources of infection may include flies which act as vectors and carry the spores of *L. fungicola* from infected mushrooms (Ware, 1933; Tibbles et al., 2005; Shamshad et al., 2009). The spores of *L. fungicola* are covered in a sticky mucilage which allows them to adhere to pests such as *Lycoriella ingenua* and *Bradysia ocellaris* (Shamshad et al., 2009). Contaminated equipment and dust/debris from the growing rooms and surrounding areas have also been shown to aid the spread of *L. fungicola* spores around mushroom farms (Grogan, 2001; Largeteau et al., 2004). The spores of *L. fungicola* will not survive at temperatures higher than 40 °C, therefore the compost in which *A. bisporus* mycelium grow and develop can be ruled out as a primary source of infection due to the high temperatures it is exposed to.

A primary infection of *L. fungicola* results in small undifferentiated masses or ‘bubbles’ of *A. bisporus*. A primary infection occurs when the mushroom pins are infected, and if left untreated they will develop into large, undifferentiated masses of mushroom tissue. Spores from these primary infections are produced and may be dispersed by water splash and/or flies. If they land on other mushrooms on the beds it results in the development of brown spots. Stipe blow out results in a splitting of the stalk tissue and is common in heavily diseased crops and results in grossly deformed mushrooms (Berendsen et al., 2010; North & Wuest, 1993). The germination of *L. fungicola* spores is inhibited by the presence of microbiota within the soil, this is referred to as soil fungistasis (Lockwood & Filonow, 1981). This inhibition is annulled by *A. bisporus* mycelium which provides nutrients such as carbon for *L. fungicola* spore germination (Carrasco et al., 2019).

Mushroom growers can limit the presence of *L. fungicola* on their crops through strict hygiene control methods and the use of chemical fungicides. For the past few decades, fungicides have provided good protection against this pathogen. However, complete control of this disease has proven difficult due to fungicide resistance and the increasing number of fungicides which are being phased out by various governmental and environmental agencies. Integrated pest management (IPM) strategies are promoted under the Sustainable Use of Pesticides Directive (SUD) 2009/128/EC (Anon, 2009) which states that the use of chemical fungicides should be avoided where possible as they can be harmful to both human and environmental health. Many strains of *L. fungicola* have also developed resistance or tolerance to fungicides over the years, such as the benzimidazoles and prochloraz-manganese which are commonly used to prevent their growth (Gea et al., 2005; Gea et al., 2021; Grogan, 2008). There is an urgent need to identify alternative treatment options to control mushroom diseases. SERENADE® (AgraQuest Inc.) (*B. velezensis* strain QST 713) and Serifel (*Bacillus amyloliquefaciens* strain MBI 600) are commercially available biocontrol agents which have shown potential to control *L. fungicola* growth (Stanojević et al., 2019). *B. velezensis* QST 713 has been shown to produce biofilms and antimicrobial compounds as bio-protection strategy against *T. aggressivium* (Marrone, 2002; Pandin et al., 2019). However, due the low number of biocontrol options commercially available, there is a need to identify more species which may be used as biocontrol agents in the future.

*B. velezensis* (strain Kos) is a newly identified bacterial species which was isolated from mushroom casing by Kosanovic et al.*,* (2021) and was shown to be inhibitory towards *Trichoderma aggressivum* which causes green mould disease (Kosanovic et al., 2021) and *Cladobotryum mycophilum* which causes cobweb disease (Clarke et al., 2022). It was also shown that this strain does not negatively impact *Agaricus bisporus*. This strain was named *B. velezensis* R8.3 during initial studies by Kosanovic et al., (2021) but was then renamed to *B. velezensis* (Kos) for this work. The aim of this work is to characterise the impact of two strains of *B. velezensis* (QST 713 & Kos) on the growth and proteomic response of *L. fungicola* in vitro*.*

## Materials and methods

### Culture conditions

*L. fungicola* (Teagasc isolate: 1722) was isolated from an infected mushroom crop and was stored in a culture collection located at Teagasc Research Centre, Ashtown (Dublin, Ireland)*.* The *L. fungicola* cultures were grown and maintained on potato dextrose agar (PDA) (Biokar diagnostics) at 25 °C for 5 days, in the dark. Liquid cultures of *L. fungicola* were grown in Sabouraud dextrose liquid broth (SDB) (Oxoid) for 48 h at 25 °C and 120 rpm. *B. velezensis* (strain Kos) was originally isolated from mushroom compost by Kosanovic et al.*,* (2021). The strain was obtained from liquid nitrogen stocks at Maynooth University (Kildare, Ireland) and was grown on nutrient agar (NA) plates (Oxoid) in the dark at 25 °C for 3 days.

*B. velezensis* QST 713, the active strain in the commercial product SERENADE® was used during this work. *B. velezensis* QST 713 was grown and maintained on NA at 25 °C for 3 days.

### Collection of bacterial culture filtrate

Liquid cultures of *B. velezensis,* strain QST 713 and Kos were grown in 50 ml nutrient broth (NB) (Oxoid) at 30 °C, 120 rpm, in the dark in an orbital incubator. At various time points (24, 48, 72 and 96 h), flasks were removed from the incubator and the culture filtrate (CF) from the bacterial cultures were collected through centrifugation (1000 x g, 20 min). CF was filtered through a 0.45 μm filtropur S filters (Sarstedt Ltd). CF stocks from the bacterial strains were kept frozen at −20 °C until required.

### The effect of *B. velezensis* culture filtrate and cells on the growth of *L. fungicola* in vitro

A conidial suspension of *L. fungicola* was prepared and adjusted to ×10^5^/ml using a haemocytometer. Aliquots (100 μl) of *L. fungicola* were spread onto PDA plates (×10^4^/plate) using a sterile spreader. The plates were left to dry for 15 min before adding wells (8 mm diameter) to the PDA. Culture filtrates (50 μl) of *B. velezensis*, strain QST 713 and Kos isolated at 24, 48, 72 and 96 h were added to individual wells in triplicate. Plates were incubated at 25 °C, in the dark.

Cell culture drops (10 μl) of *B. velezensis* strain QST 713 and Kos from 24, 48, 72 and 96 h cultures were applied directly onto PDA which contained *L. fungicola* (×10^4^/plate). Plates were incubated at 25 °C, in the dark.

The density of a spore suspension of *L. fungicola* (1 × 10^6^/ml) was ascertained using a haemocytometer and 1 ml of this was added to SDB (50 ml) to give a final spore density of 2 × 10^4^/ml. Liquid cultures of *L. fungicola* were grown for 48 h at 25 °C and 120 rpm*. L. fungicola* cultures were supplemented with either NB (control) (25% *v*/*v*), 96 h *B. velezensis* (Kos) CF (25% *v*/*v*) or 96 h *B. velezensis* (QST 713) CF (25% *v*/*v*). Five replicates were used per treatment. *B. velezensis* (Kos) 96 h CF was chosen to proceed with for further experiments as previous work has shown that this time point results in the largest zone of inhibition (Clarke et al., 2022). Cultures were grown under the same conditions for a further 24 h. The mycelia within the flasks were separated from the liquid supernatant using Miracloth (Merck). The wet weight (g) of the mycelium in each treatment was then determined.

### Fluorescent and scanning electron microscopy

A small sample of *L. fungicola* hyphae from each of the liquid culture treatments (*L. fungicola* treated with either NB (A), 96 h *B. velezensis* (QST 713) (B) or 96 h *B. velezensis* (Kos) CF (C)) was collected and applied to the centre of a glass microscopic slide. The hyphae were washed three times with PBS (50 μl). Calcofluor white (25 μl, Sigma-Aldrich) was applied for 5 min to stain the hyphae and the stain was removed by washing with PBS once more. A glass cover slip was then placed on top of the samples and the hyphae were imaged using an Olympus BX51 fluorescent microscope (X40 lens).

The hyphae were visualised using a scanning electron microscope (SEM). A droplet of the PBS washed hyphae from each treatment was placed onto a sterilised microscopic cover slip. Samples were fixed to the slide with 5% (*v*/*w*) glutaraldehyde (Sigma-Aldrich) for 2 h, unadhered cells were removed by gentle washing with pre-warmed PBS. Slides were subjected to sequential washing with increasing ethanol concentrations (35, 50, 70, 80, 90 and 100%) to facilitate dehydration. The samples were treated with hexamethyldisilazane (Sigma-Aldrich) and air-dried overnight. Samples were sputtered with gold (6–12 nm) prior to imaging. Hyphae were imaged using a HITACHI S-3200 N Scanning electron microscope (X500 lens).

### Label free quantitative proteomics of *L. fungicola* treated with *B. velezensis* culture filtrates

Proteins were extracted from *L. fungicola* mycelium that had been treated with either 96 h *B. velezensis* (QST 713) CF or 96 h *B. velezensis* (Kos) CF. *L. fungicola* cultures were grown for 48 h before being supplemented with either treatment (25% *v*/*v*). *L. fungicola* cultures supplemented with NB (25% *v*/*v*) were used as a control. Fungal hyphae were crushed in liquid nitrogen using a pestle and mortar. Cell lysis buffer (8 M urea, 2 M thiourea, and 0.1 M Tris-HCl (pH 8.0) dissolved in 25 ml ddH_2_O) which had been supplemented with various protease inhibitors (leupeptin, pepstatin A and Phenylmethylsulfonyl fluoride (PMSF) (10 μg/ml)) was applied to the crushed hyphae to collect the cell lysate. The protocol for protein extraction and mass spectrometry sample preparation is described in Margalit et al.*,* (2020).

Samples were analysed on a QExactive (ThermoFisher Scientific, USA) high resolution accurate mass spectrometer connected to a Dionex Ultimate 3000 (RSLCnano) chromatography system. Peptide mix (0.75 μg) was applied to the QExactive. Peptides were separated by an increasing acetonitrile gradient from 2%–40% on a Biobasic C18 Picofrit column (100 mm length, 75 mm ID), using a 120-min reverse phase gradient at a low rate of 250 nl/min. A full MS scan of range 200–2000 was followed to select the 15 most intense ions prior to MS/MS.

Quantitative analysis of the data generated from the QExactive run was preformed using Andromeda search engine in Max-Quant (version 1.6.17 https://www.maxquant.org/). Max-Quant was used to identify the proteins within the sample and then correlate them against a *Trichoderma reesei* proteome fasta file (Proteome ID: UP000024376, Genome accession: #JABP01000000) downloaded from www.uniprot.org. There is no *L. fungicola* proteome database available on UniProt therefore a closely related species, *T. reesei* was chosen to analyse the data.

Perseus (version 1.6.14.0) was used for data analysis and graphical generation (Margalit et al., 2020). The LFQ intensities were log2-transformed and filtered to remove proteins with non-existent values which suggest absence or low abundance within the sample. A principal component analysis (PCA) was used to group the data sets based on their similarities. Gene ontology (GO) mapping was carried out using a Blast 2 Go tool (https://www.blast2go.com/). The UniProt gene IDs from all of the proteins within the Perseus dataset were run against a *Trichoderma reesei* fasta file. The GO file which resulted was then uploaded to Perseus to provide terms for gene ontology biological process (BP), gene ontology cellular component (CC), gene ontology molecular function (MF) and UniProt name for each protein identified. Multiple sample t-tests and ANOVA significance tests were used to identify the statistically significant and differentially abundant (SSDA) proteins. The SSDA proteins with a relative fold change greater than ± 0.58 were retained for analysis. These proteins were then used to make volcano plots by plotting the log_2_ fold change on the x axis against the log p values on the y axis for each pairwise comparison. SSDA proteins were also Z- score normalised and used for the generation of a heatmap and hierarchical clustering.

The SSDAs for both *B. velezensis* (QST 713) and *B. velezensis* (Kos) samples were run through a Omicsbox software tool (v. 2.0.10) to preform GO mapping. The mass spectrometry proteomics data have been deposited to the ProteomeXchange Consortium via the PRIDE [1] partner repository with the dataset identifier PXD028506.

## Results

### The effect of *B. velezensis* (QST 713) and *B. velezensis* (Kos) culture filtrate on the growth of *L. fungicola* in vitro

*L. fungicola* plate cultures treated with *B. velezensis* (QST 713 and Kos) culture filtrate from various timepoints were analysed after 72 h and there were no zones of inhibition detected for either treatment. This would suggest that the culture filtrate from *B. velezensis* (Kos) and *B. velezensis* (QST 713) are not capable of inhibiting the growth of *L. fungicola* on plate cultures (Fig. S1A). However, zones of clearance were identified around the areas on the plate where *B. velezensis* (QST 713) and *B. velezensis* (Kos) cell culture drops were applied indicating that both bacterial cell cultures could inhibit the growth of *L. fungicola* (Fig. S1B)*.*

**Fig. 1:**
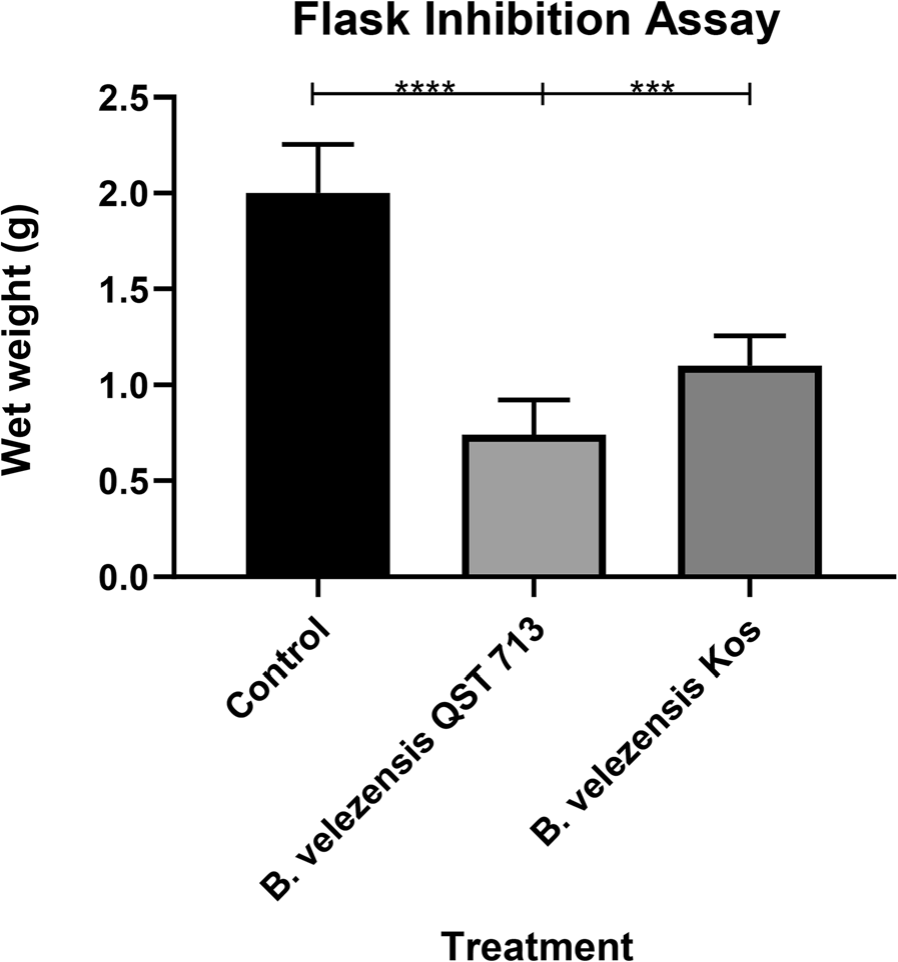
*Lecanicillium fungicola* liquid cultures (*x*10^4^/ml) were grown in SDB and were supplemented with either 12.5% *v*/*v* NB (control), 12.5% *v*/*v* 96 h *Bacillus velezensis* (QST 713) CF or 12.5% *v*/*v* 96 h *B. velezensis* (Kos) CF. Wet weight measurements of 5 replicates per treatment were recorded. Average wet weight for each treatment is displayed above, Error bars represent standard deviation. **** = <0.0001 *** = 0.0002

Liquid cultures of *L. fungicola* treated with either NB (control), 25% *v*/*v B. velezensis* (Kos) 96 h CF or 25% *v*/*v B. velezensis* (QST 713) 96 h CF were analysed after 24 h. The wet weight of the mycelium within each flask was recorded. The control flasks had an average wet weight of 2 ± 0.22 g. The mycelium within the *B. velezensis* (QST 713) treated flasks weighed an average of 0.74 ± 0.16 g and *B. velezensis* (Kos) treated flasks weighed 1.1 ± 0.14 g. This represents a percentage biomass decrease of 63% for *B. velezensis* (QST 713) (*P* < 0.0001) and 45% for *B. velezensis* (Kos) treatment ((*P* < 0.0002) (Fig. 1)*.*

### Fluorescent and scanning electron microscopy

**Fig. 2:**
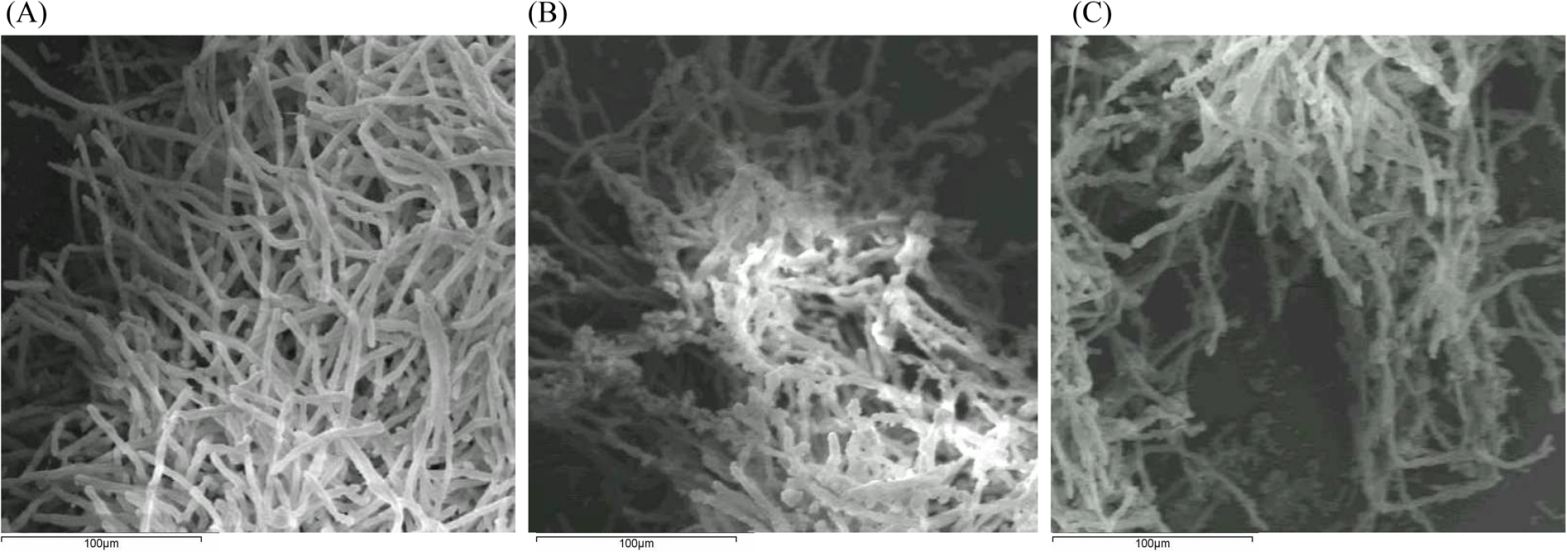
*Lecanicillium fungicola* cultures (*x*10^4^/ml) grown for 48 h at 25 °C and then grown for a further 24 h when supplemented with either; **a** 25% *v*/*v* NB. **b** 25% *v*/*v* 96 h *Bacillus velezensis* (QST 713) CF. **c** 25% *v*/*v* 96 h *B. velezensis* (Kos) CF. Hyphae from each treatment were collected and imaged on an HITACHI S-3200 N Scanning electron microscope at magnification X500

The effect of *B. velezensis* (QST 713) and *B. velezensis* (Kos) 96 h CF (25% *v*/*v*) on *L. fungicola* hyphae was visualised after 24 h. The SEM images show a clear difference between the hyphae taken from control flasks, from those taken from *B. velezensis* (QST 713) and *B. velezensis* (Kos) treated flasks. The hyphae from the control (treated with NB only) appear to be cylindrical and regular in shape (Fig. 2a). This is in contrast to the *B. velezensis* (QST 713) and *B. velezensis* (Kos) CF treated hyphae which appear irregular, rounded, and deformed (Fig. 2b, c). This indicates that *L. fungicola* hyphae are disrupted when either *B. velezensis* (QST 713) or *B. velezensis* (Kos) CF is present. The fluorescent images also show similar damage to *B. velezensis* (QST 713) and *B. velezensis* (Kos) treated hyphae (Fig. S2). The *Bacillus*-treated hyphae appear to be irregular and damaged compared to the control hyphae.

### Label free quantitative proteomics of *L. fungicola* treated with *B. velezensis* culture filtrates

**Fig. 3:**
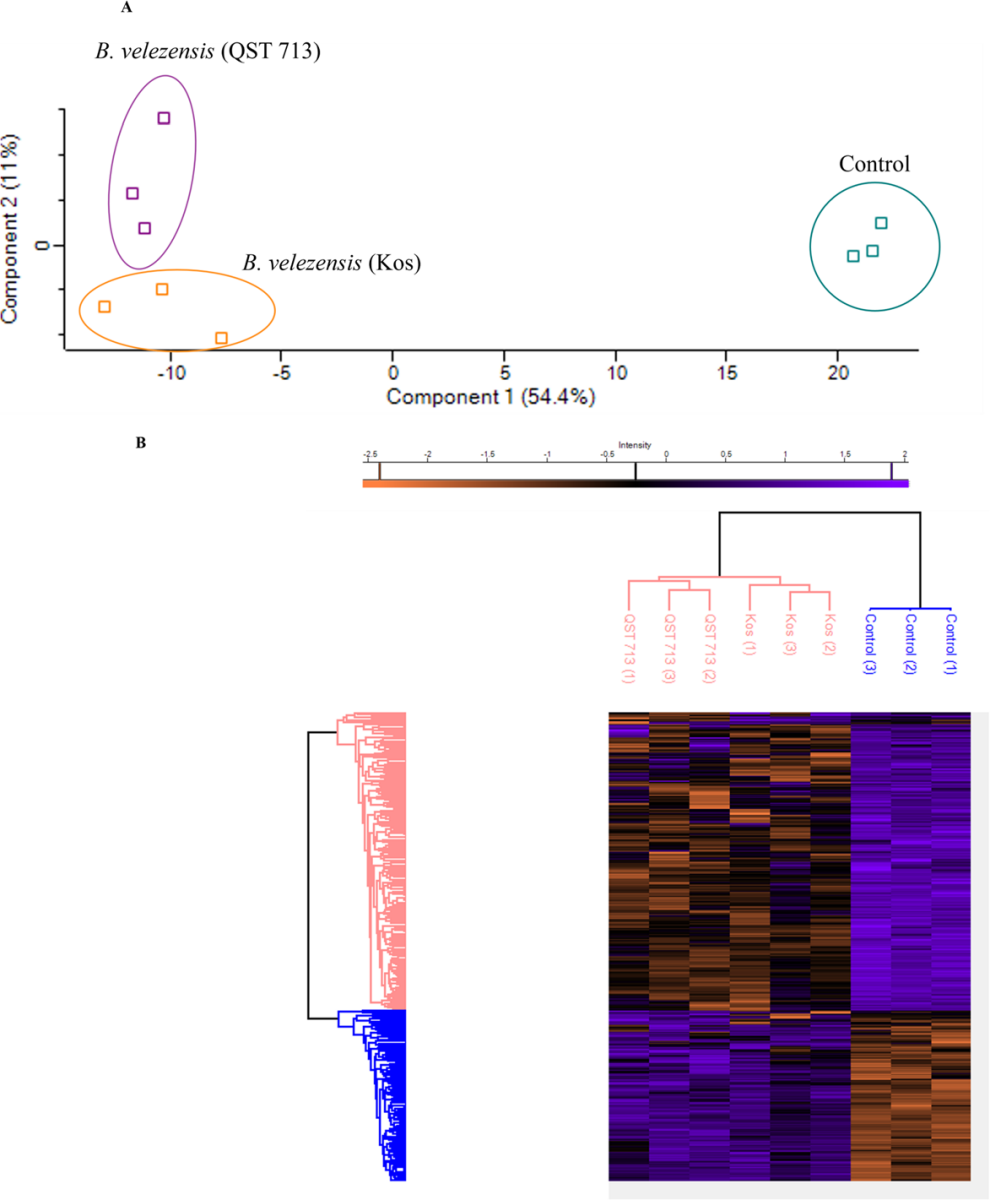
**a** PCA grouping the samples based on similarities within their proteome. Control samples (blue) clustered together and were distanced away from the *Bacillus velezensis* (QST 713) (purple) and *B. velezensis* (Kos) (orange) samples. *B. velezensis* (QST 713) and *B. velezensis* (Kos) samples clustered close together but remained separate from one another. **b** Hierarchical clustering separates the control samples from the *B. velezensis* (QST 713) and *B. velezensis* (Kos) samples on separate lineages. The heat map pattern also indicates that in areas of increased protein abundance in control samples (purple) there is decreased protein abundance in *B. velezensis* (QST 713) and *B. velezensis* (Kos) (orange)

The whole cell proteomic response of *L. fungicola* when exposed to *B. velezensis* (Kos) 96 h CF (25% *v*/*v*) and *B. velezensis* (QST 713) 96 h CF (25% *v*/*v*) was investigated using label free quantitative (LFQ) proteomics. A total of 1962 proteins were initially identified using Perseus (v 1.6.14.0). This number was reduced to 866 after various filtration steps. A PCA was generated with the resulting data set. Samples with similar proteomes will cluster together on a PCA. The PCA groups the control samples together and distances them away from the two sets of treatment samples. It grouped the samples treated with *B. velezensis* (QST 713) and *B. velezensis* (Kos) CF close together but still remaining separate from one another (Fig. 3a). Hierarchal clustering places the control samples on a separate lineage to either *B. velezensis* (QST 713) or *B. velezensis* (Kos) treatment samples. The pattern on the heat map shows that in areas indicating an increase in protein abundance in the treatment samples, there was a corresponding decreased relative protein abundance for the control samples (Fig. 3b).

**Fig. 4:**
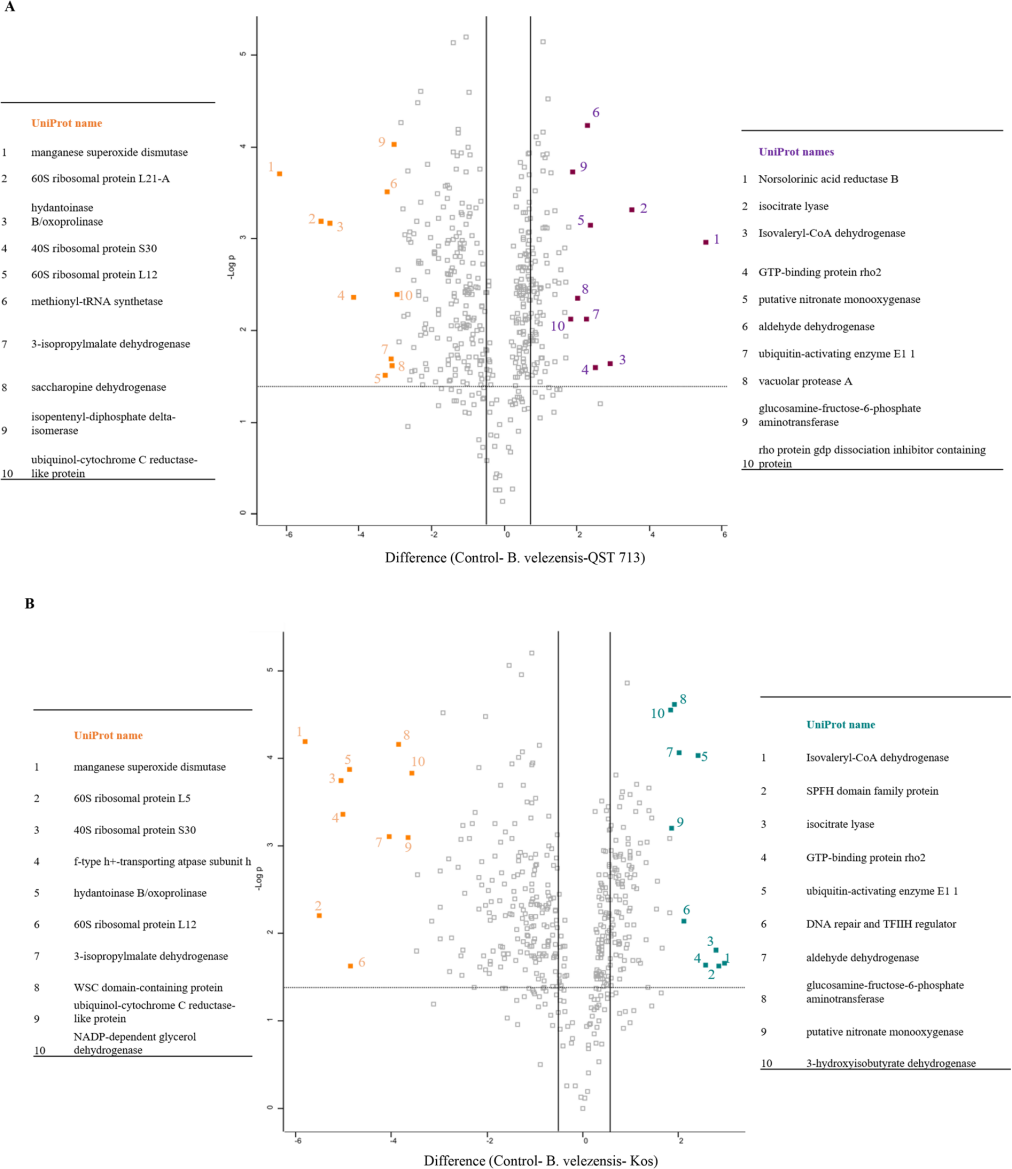
Volcano plots display the distribution of statistically significant and differentially abundant (SSDA) proteins which have a -log p fold change >1.3 and difference > ± 0.58 within either Control/ *Bacillus velezensis* (QST 713) **a** or Control/*B. velezensis* (Kos) **b** treatment groups

Volcano plots show the distribution of SSDA proteins in *B. velezensis* (QST 713) and *B. velezensis* (Kos) treated samples compared to the control (Fig. 4). A total of 328 SSDA proteins were identified in *B. velezensis* (QST 713) treated samples (129 increased and 199 decreased in abundance, Fig. 4a). SSDA proteins which had increased in abundance with the highest fold change included norsolorinic acid reductase B (47-fold), isocitrate lyase (11-fold) and isovaleryl-CoA dehydrogenase (8-fold). The proteins which were decreased at the highest fold change included; manganese superoxide dismutase (−73-fold), 60S ribosomal protein L21-A (−32-fold) and 40S ribosomal protein S30 (−17-fold) (Supplementary Table 1–2).

A total of 288 SSDA proteins were identified in *B. velezensis* (Kos) treated samples (103 increased and 185 decreased in abundance, Fig. 4b). Many of the SSDAs identified were the same proteins identified as significant for the *B. velezensis* (QST 713) treated samples. Isovaleryl-CoA dehydrogenase (8-fold) and isocitrate lyase (7-fold) were also increased in *B. velezensis* (Kos) treated samples as well as SPFH domain family protein (7-fold) and MMS19 nucleotide excision repair protein (4-fold). Manganese superoxide dismutase (−56-fold), 60S ribosomal protein L5 (−45-fold) and 40S ribosomal protein S30 (−33-fold) were also reduced in abundance in *B. velezensis* (Kos) treated samples as well as NADP-dependent glycerol dehydrogenase (−12fold) (Supplementary Table 3–4). There was a total of 263 common SSDA proteins shared between the *B. velezensis* (QST 713), and *B. velezensis* (Kos) treatments, meaning there were 65 SSDA proteins exclusive to *B. velezensis* (QST 713) and 25 proteins exclusive for *B. velezensis* (Kos) treatment. The SSDAs with the highest fold change in either *B. velezensis* (Kos) or *B. velezensis* (QST 713) treated *L. fungicola* samples are listed in supplementary Table 1–4.

Omicsbox GO mapping shows that biological processes like translation and peptide biosynthetic process are reduced in *B. velezensis* (Kos) and *B. velezensis* (QST 713) treated samples. It also indicates that biological process like glutamine family amino acid metabolic process and molecular functions like isocitrate lyase activity are increased in abundance in *B. velezensis* (QST 713) and *B. velezensis* (Kos) samples. Proteasomal protein catabolism and proteolysis are listed as increased in *B. velezensis* (QST 713) treated samples, while D-threo-aldose-1-dehydrogenase (oxidoreductase) activity is increased in *B. velezensis* (Kos) treated samples (Fig. S3).

## Discussion

This work highlights the in vitro growth inhibition and proteomic response of *L. fungicola* to *B. velezensis* (QST 713), present in the commercially available biocontrol product, Serenade ®, and to a newly isolated *B. velezensis* (Kos) strain. The aim was to establish how similar these two strains were to each other and whether they were inhibiting the growth of *L. fungicola* in a similar manner.

The results of the plate inhibition assay showed that cell culture drops from *B. velezensis* (QST 713) and *B. velezensis* (Kos) cells produced zones of inhibition on *L. fungicola* plate cultures. The CF of *B. velezensis* (QST 713) and *B. velezensis* (Kos) were both able to inhibit *L. fungicola* biomass accumulation in liquid cultures. The bacterial CF was able to inhibit growth of *L. fungicola* in liquid cultures, but not in plate cultures. Kosanovic et al.*,* (2021) found a similar result when testing *B. velezensis* (Kos) against *T. aggressivium*. In previous work, it was demonstrated that this CF is capable of inhibiting the growth of *C. mycophilum* on plates and this appears to be the only case of plate inhibition from this *B. velezensis* (Kos) strain (Clarke et al., 2022). It is possible that the CF was unable to inhibit the growth of *L. fungicola* in the plate inhibition assay due to the physical difference between PDA and SDB. In a liquid broth, the *L. fungicola* cells are emerged and surrounded entirely by the CF which may make them more vulnerable to inhibition. On a plate culture, it is possible that the CF may dissolve partially into the agar under the surface of the *L. fungicola* and may not interact fully with the pathogen. SEM imaging of hyphae treated with the bacterial CF also confirmed that *B. velezensis* (QST 713) and *B. velezensis* (Kos) CF disrupted the *L. fungicola* hyphae. The growth of *L. fungicola* is stunted in the presence of *B. velezensis* and this results in irregular, shorted hyphae which contrast with the healthy control hyphae.

Proteomic analysis further supports the finding that *B. velezensis* (QST 713) 96 h CF causes significant growth inhibition and stress to *L. fungicola*. The proteomics results also suggest that *B. velezensis* (Kos) is inhibiting *L. fungicola* in a similar way to the *B. velezensis* (QST 713) strain. The PCA, hierarchical clustering and heatmap all point to differences between the proteome of *L. fungicola* samples treated with either *B. velezensis* (QST 713) or *B. velezensis* (Kos) 96 h CF compared to the control samples. Clearly, *B. velezensis* is inducing an abnormal *L. fungicola* proteomic response. The high degree of separation between treatment and control samples in the PCA indicates that the proteome of *B. velezensis* (QST 713) and *B. velezensis* (Kos) treated samples differ to the control samples. The PCA groups the *B. velezensis* (QST 713) and *B. velezensis* (Kos) samples close together, indicating that they have a similar impact on the proteome of *L. fungicola* suggesting that they are working in a similar way. This finding is reflected in the separation between control and treatment samples, and similarities between the two treatment samples highlighted in hierarchical clustering and the heat map.

The proteomic response of *L. fungicola* against *B. velezensis* (QST 713) and *B. velezensis* (Kos) is similar as there was a high proportion of shared SSDA proteins between them. This suggests that similar activities are been triggered or reduced in response to both treatments. *B. velezensis* (QST 713) had a higher number of SSDA and had more SSDAs which were exclusive. This may indicate that *B. velezensis* (QST 713) is inducing a greater proteomic response from *L. fungicola*. This would support the results from the flask inhibition assay in which *B. velezensis* (QST 713) resulted in a larger biomass reduction compared to *B. velezensis* (Kos). This indicates that although both strains are working in a similar manner, *B. velezensis* (QST 713) appears to be better at inhibiting the growth of *L. fungicola* in vitro. The SSDAs which statistically increased in abundance for both treatments were involved in processes such as oxidoreductase activity, ubiquitination and DNA repair which are all associated with an oxidative stress response. Isocitrate lysate was also increased in abundance in both treatments. This enzyme is involved in the glyoxylate cycle, which acts as a variant of the tricarboxylic acid cycle. The glyoxylate cycle has been shown to be required for fungal virulence and stress response (Lorenz & Fink, 2001). The majority of SSDAs which were statistically significantly decreased in abundance for both treatments were involved in growth process and translation*. L. fungicola* may be reducing processes like growth to conserve energy to maintain a stress response against the *B. velezensis* strains. Manganese superoxide dismutase had the largest protein fold decrease in both *B. velezensis* (QST 713) (−73-fold) and *B. velezensis* (Kos) (−55-fold) treated samples compared to the control. Manganese superoxide dismutase has been shown to play a key role in protection in fungal species against oxidative stress (Holley et al., 2011). Its reduced abundance means that *L. fungicola* from these samples has a reduced antioxidant ability to protect itself from the stress initiated by the *B. velezensis* strains. Omicsbox GO mapping also confirms that activities associated with growth are reduced in *B. velezensis* treated samples, while activities associated with stress are increased.

The continuous application and reliance on chemical fungicides to treat mushroom diseases has resulted in reduced sensitivity and resistant strains (Bollen & Zaayen, 1975; Gea et al., 1996; Gea et al., 2021). Developing non-chemical treatment methods is listed in the eight principals of IPM as outlined in EU Directive 2009/128/EC (Anon, 2009). Unfortunately, in the field the level of protection achieved by biocontrol agents is often inferior compared to fungicides and there is an understandable hesitation to adopt this approach. Biocontrol treatment alone may not be an adequate replacement for fungicides. However, combining biocontrol treatments with other strategies of IPM (Prevention and suppression, monitoring and decision making) would reduce our reliance on chemical fungicides (Barzman et al., 2015). As *Bacillus* species are found naturally in mushroom casing and are generally regarded as safe (GRAS) (Borriss, 2015), it would also be a more environmentally friendly option.

Both the in vitro growth assays and proteomic analysis have demonstrated that *B. velezensis* (QST 713) can reduce the growth of *L. fungicola* and induce a stress response in the pathogen. To the best of our knowledge, this is the first time the proteomic response of *L. fungicola* to this important biocontrol strain has been published. We have also shown that the newly identified *B. velezensis* (Kos) is working in a similar manner to *B. velezensis* (QST 713), which is already approved for use on many crops. Previous work has also demonstrated that *B. velezensis* (Kos) can inhibit both cobweb disease (*C. mycophilum*) and green mould disease (*T. aggressivium*) in vitro (Clarke et al., 2022; Kosanovic et al., 2021). The results show that *B. velezensis* (QST 713) can achieve a high level of inhibition against *L. fungicola* in vitro. The results also indicate the potential of *B. velezensis* (Kos) as a new biocontrol treatment for mushroom disease control in the future. Future in vivo trials are planned to characterise the full impact of these biocontrol strains on *L. fungicola* and dry bubble disease.

## Supplementary Information

ESM 1 (DOCX 8170 kb)

ESM 2 (DOCX 25 kb)

## Abbreviations

IPM: Integrated pest management
SUD: Sustainable Use of Pesticides Directive
PDA: Potato dextrose agar
SDB: Sabouraud dextrose liquid broth
NA: Nutrient agar
NB: Nutrient broth
CF: Culture filtrate
PMSF: Phenylmethylsulfonyl fluoride
DTT: Dithiothreitol
IAA: Iodoacetamide
PCA: Principal component analysis
GO: Gene ontology
BP: Biological process
MF: Molecular function
SSDA: Statistically significant differentially abundant
LFQ: Label free quantitative-proteomic
ANOVA: Analysis of variance
