# Supplementary material for: Characterising the proteomic response of mushroom pathogen Lecanicillium fungicola to Bacillus velezensis QST 713 and Kos biocontrol agents
Source: Eur J Plant Pathol. 2022 Apr 22;163(2):369–79. doi: 10.1007/s10658-022-02482-1 (PMC9110487; doi:10.1007/s10658-022-02482-1)
Supplement: Supplementary file 1 — (DOCX 8170 kb) [file 10658_2022_2482_MOESM1_ESM.docx]

**Supplementary images:**

Supplementary fig. 1A

**
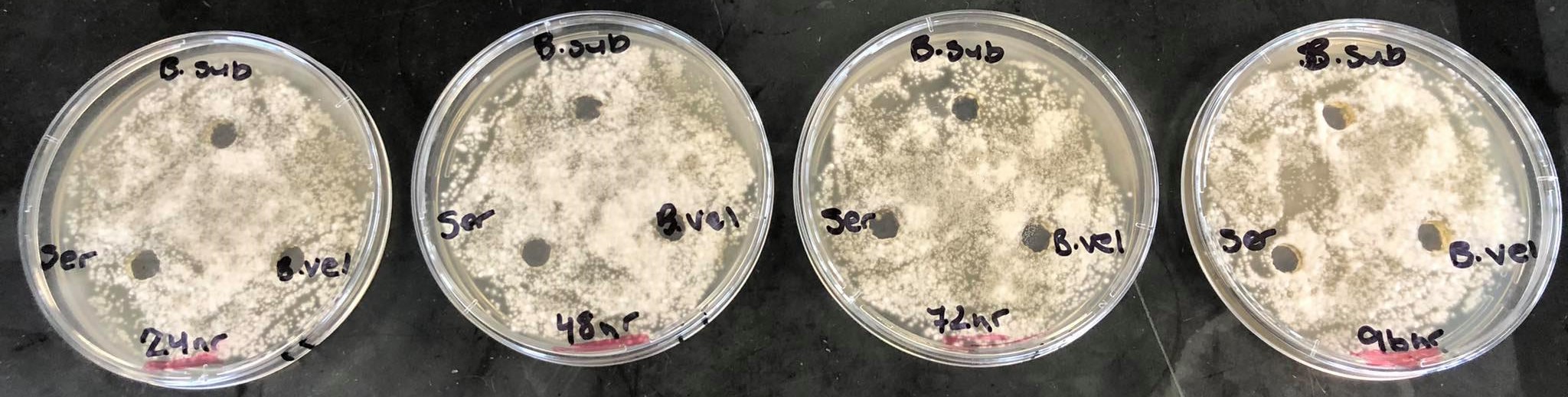
**

Supplementary. Fig 1A: No zones of inhibition present when *B. velezensis* (Kos), B. velezensis (QST 713) culture filtrate was applied against *L. fungicola* (x10^4^). (Note: *B. subtilis* depicted in this image was not included in manuscript data).

Supplementary fig. 1B


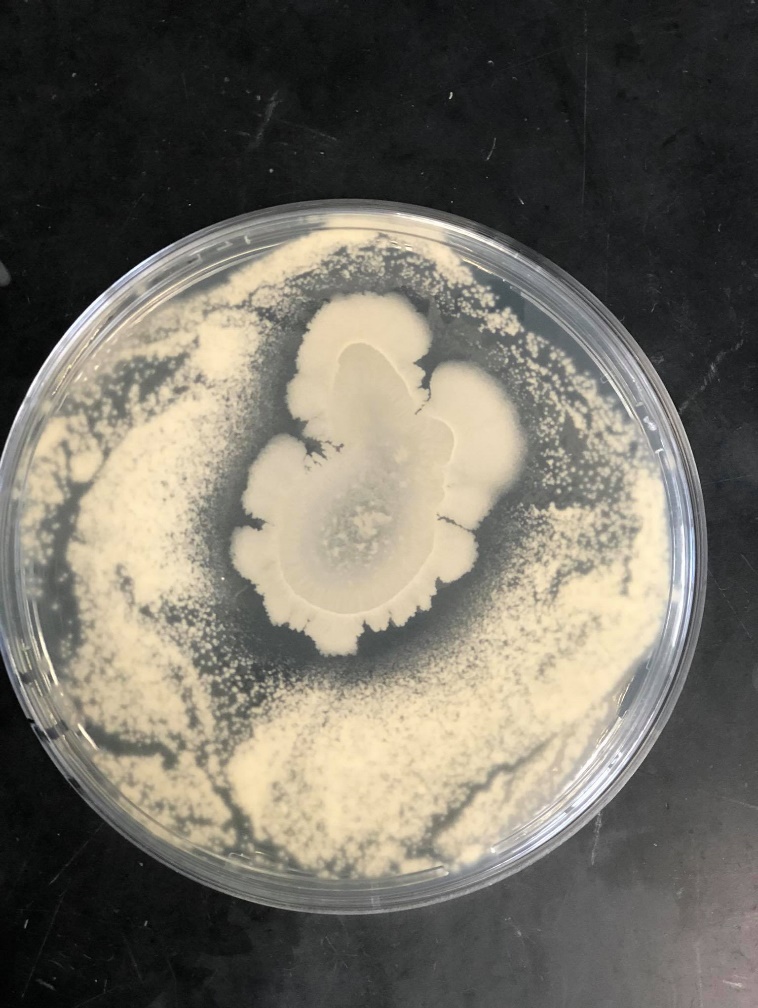

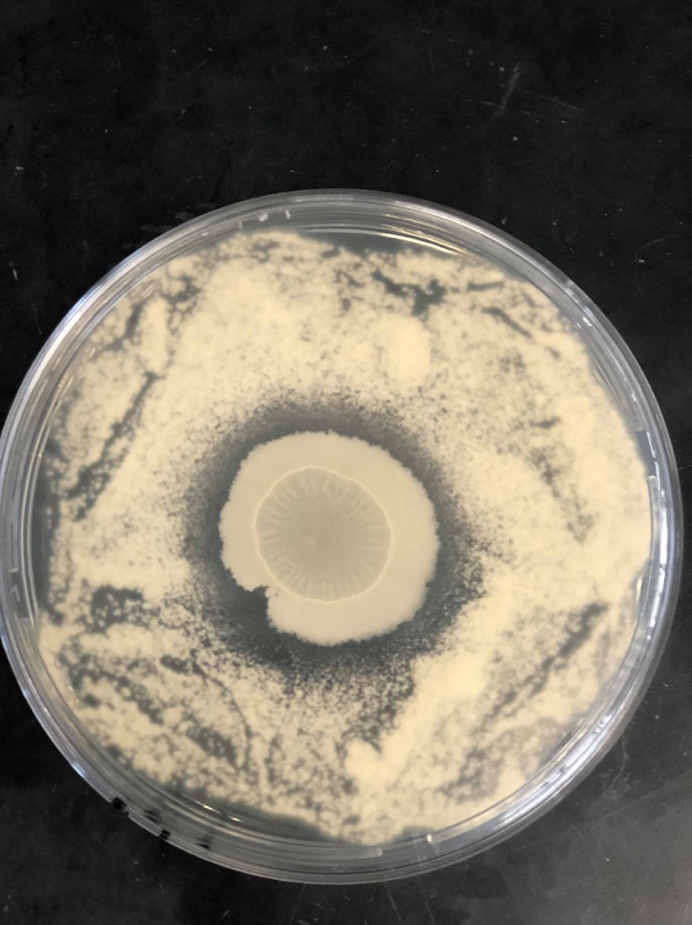


(A)

(B)

Supplementary. Fig 1B: Zones of inhibition produced by *B. velezensis* (Kos) (A) and *B. velezensis* (QST 713) (B) cells against *L. fungicola* (x10^4^).


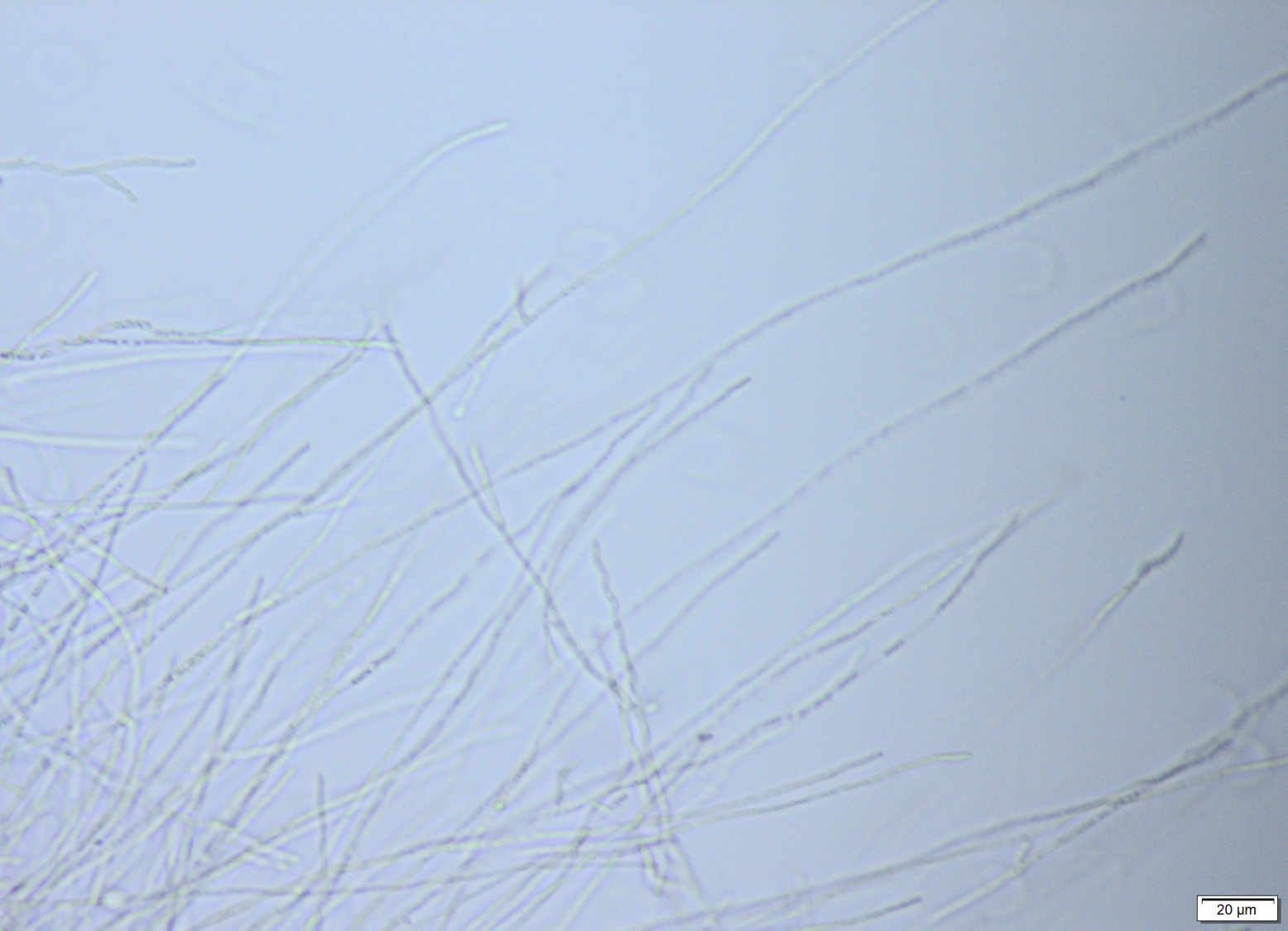


Supplementary. Fig 2

**(A)**


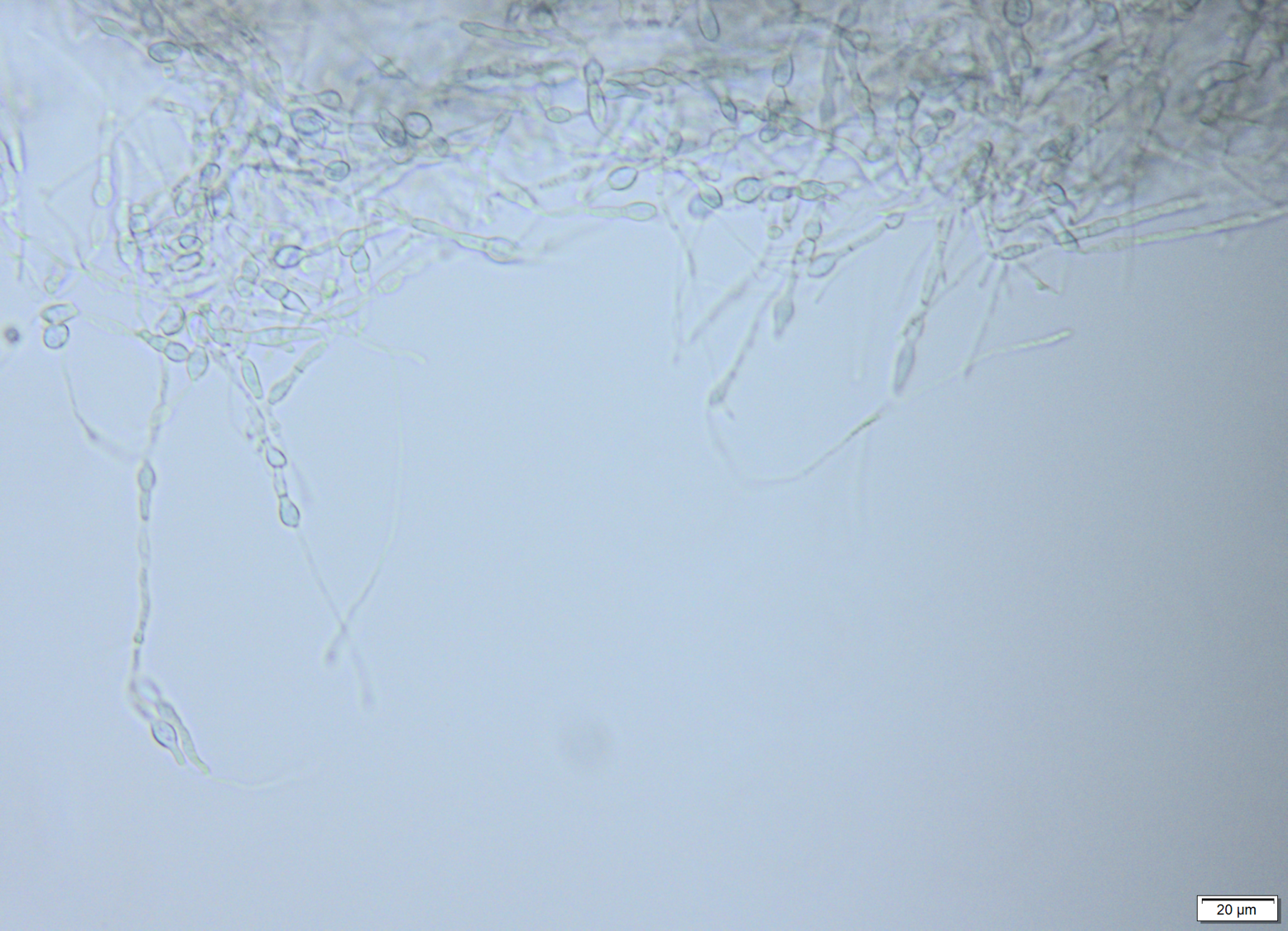


**(B)**


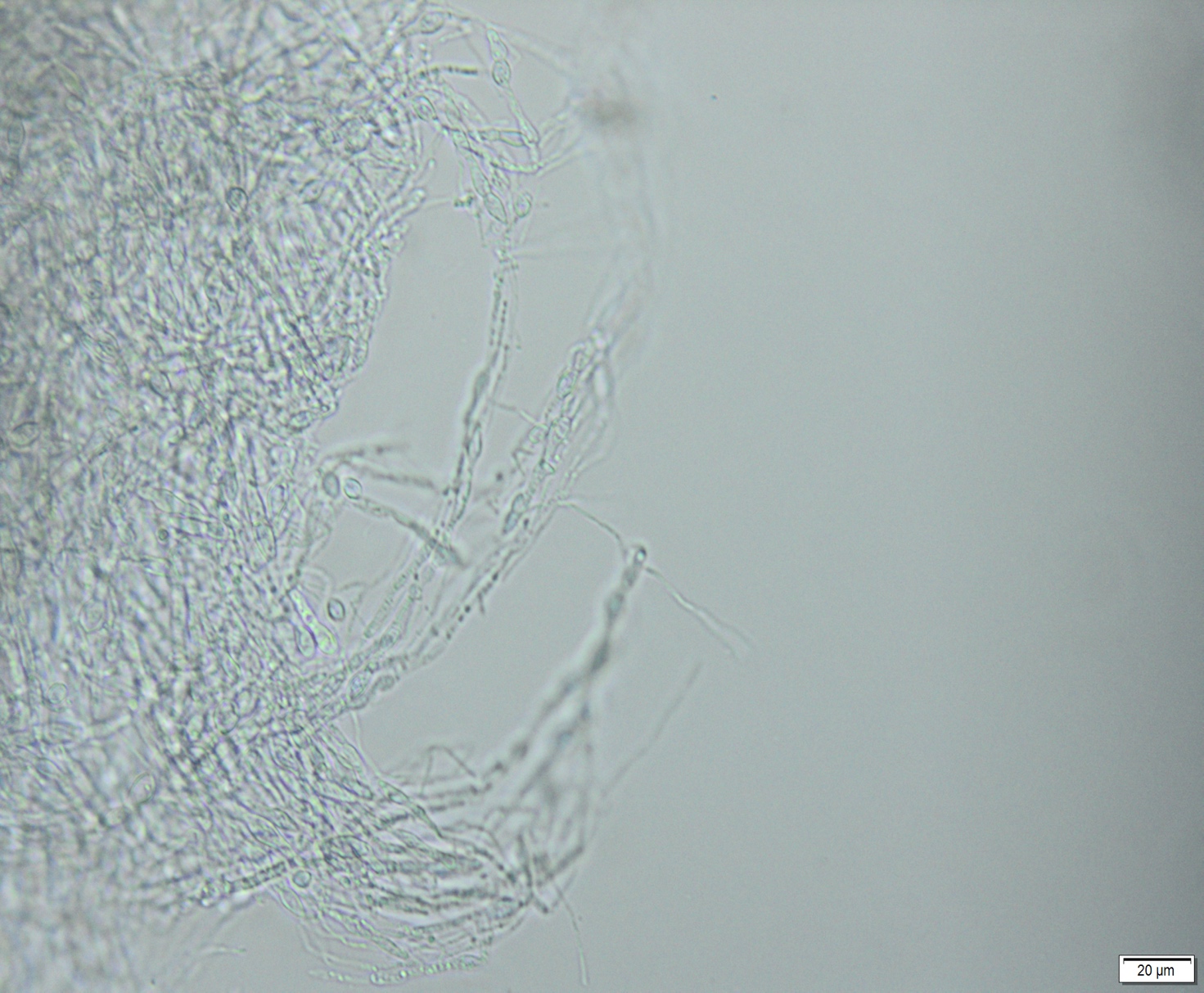


**(C)**

Supplementary. Fig 2: Florescent images taken on an Olympus BX51 fluorescent microscope (X40 lens) of *L. fungicola* hyphae treated with either NB (A), Serenade 96 hr CF (B) or *B. velezensis* 96 hr CF (C).

Supplementary. Fig 3 A


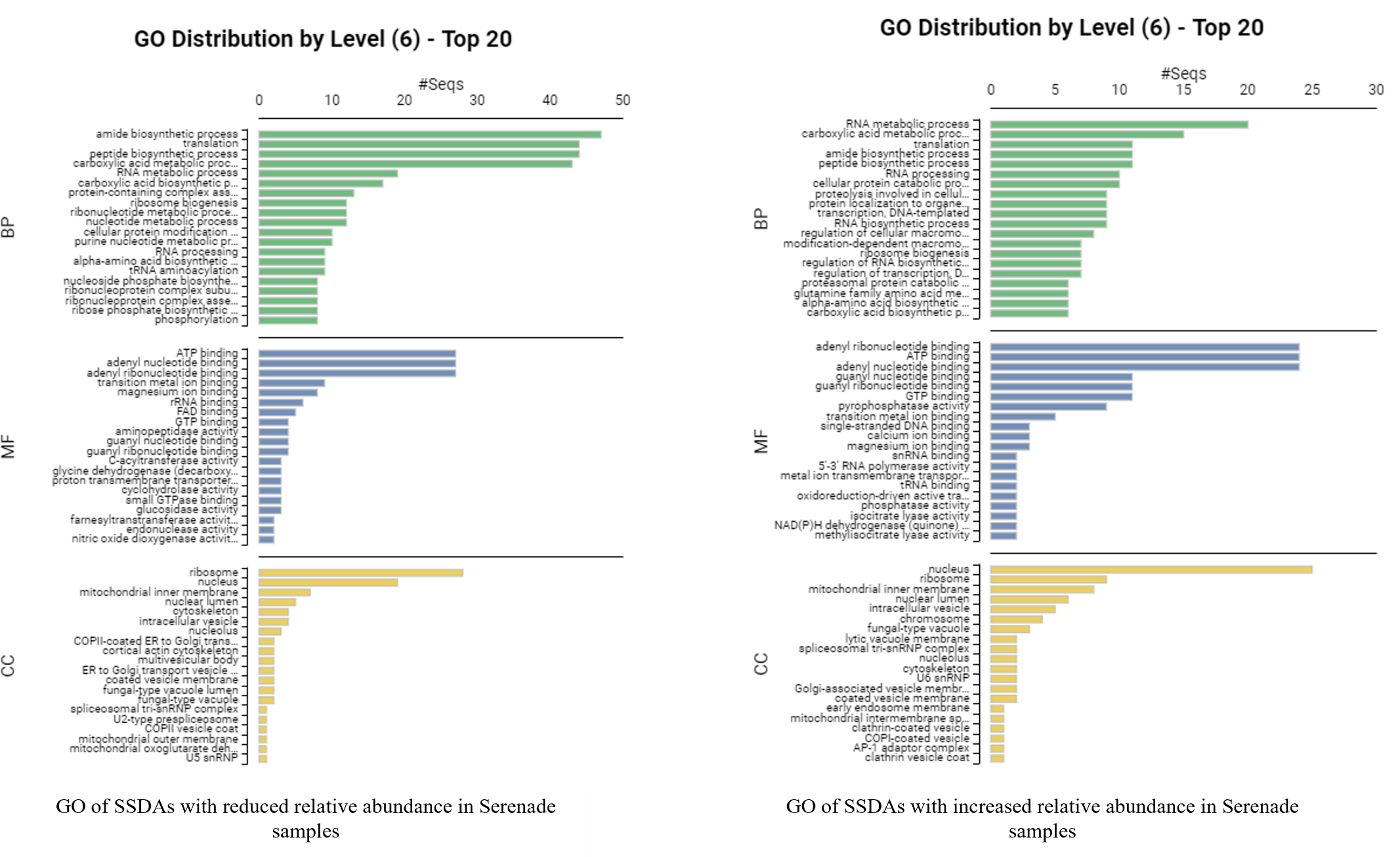


Supplementary. Fig 3A: Omicsbox gene ontology (GO) mapping of Serenade statistically significant and differentially abundant (SSDA) proteins. Graphs depict biological process (BP), molecular function (MF) and cellular component (CC).


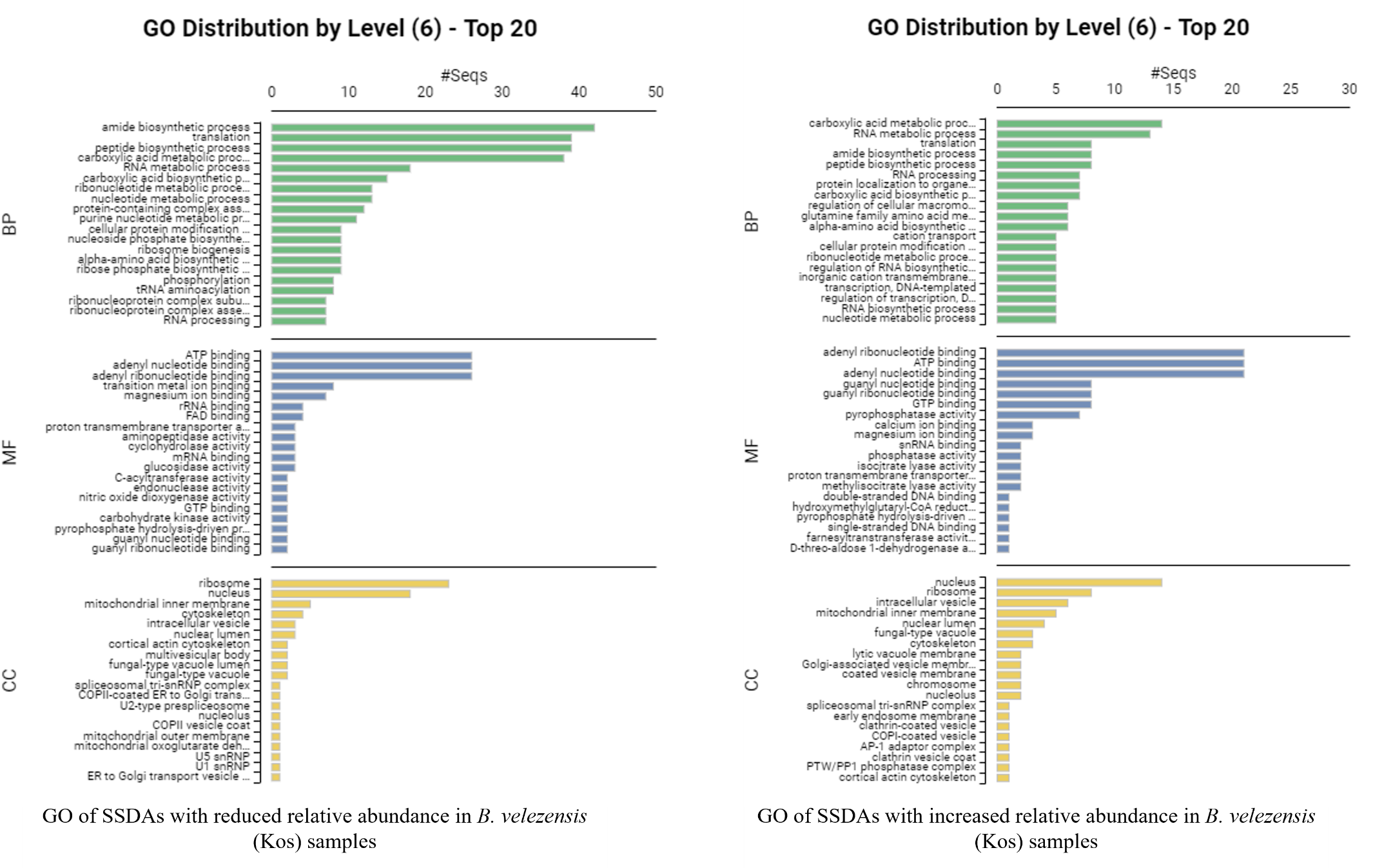
 Supplementary Fig 3 B

Supplementary. Fig 3A: Omicsbox GO mapping of *B. velezensis* (Kos) SSDA proteins. Graphs depict biological process (BP), molecular function (MF) and cellular component (CC).
