# Supplementary material for: Characterising the proteomic response of mushroom pathogen Lecanicillium fungicola to Bacillus velezensis QST 713 and Kos biocontrol agents
Source: Eur J Plant Pathol. 2022 Apr 22;163(2):369–79. doi: 10.1007/s10658-022-02482-1 (PMC9110487; doi:10.1007/s10658-022-02482-1)
Supplement: Supplementary file 2 — (DOCX 25 kb) [file 10658_2022_2482_MOESM2_ESM.docx]

| **Protein IDs** | **Actual Difference** | **Description** | **Function** |
| --- | --- | --- | --- |
| G0R973 | 47.31 | Norsolorinic acid reductase B | Oxidoreductase activity, involved in mycotoxicosis (toxic response of fungi) |
| G0RE21 | 11.37 | isocitrate lyase | carboxylic acid metabolic process |
| G0RMM8 | 7.56 | Isovaleryl-CoA dehydrogenase | acyl-CoA dehydrogenase activity |
| G0R8P2 | 5.73 | GTP-binding protein rho2 | GTPase activity, GTP binding |
| G0RU04 | 5.37 | 3-ketoacyl-CoA thiolase-like protein | Transferase activity |
| G0RAZ7 | 5.17 | putative nitronate monooxygenase | Nitronate monooxygenase activity |
| G0RKZ7 | 4.91 | Aldehyde dehydrogenase | Oxidoreductase activity |
| G0RBS0 | 4.78 | ubiquitin-activating enzyme E1 1 | ATP binding, glutathione transferase activity, antioxidant defence |
| G0RIW3 | 4.07 | Vacuolar protease A | Proteolysis |
| G0RM90 | 3.68 | glucosamine-fructose-6-phosphate aminotransferase | glutamine metabolic process (essential amino acid in states of stress) |

Supplementary Table 1: Top SSDA upregulated in Serenade treated *L. fungicola*

Supplementary Table 2: Top SSDA downregulated in Serenade treated *L. fungicola*

| **Protein IDs** | **Actual Difference** | **Description** | **Function** |
| --- | --- | --- | --- |
| G0RQS7 | 73.07 | Manganese superoxide dismutase | Removal of superoxide radicals |
| G0RGJ8 | 32.84 | 60S ribosomal protein L21-A | Translation |
| G0RTN4 | 27.68 | hydantoinase B/oxoprolinase | Hydrolase activity |
| G0RTH1 | 17.67 | 40S ribosomal protein S30 | Translation |
| G0RR73 | 9.76 | 60S ribosomal protein L12 | Translation |
| G0RQ41 | 9.34 | methionyl-tRNA synthetase | Nucleic acid-binding protein |
| G0RLM5 | 8.57 | 3-isopropylmalate dehydrogenase | magnesium ion binding, NAD binding |
| G0RTR0 | 8.46 | Saccharopine dehydrogenase | Lysine metabolism |
| G0RAZ0 | 8.19 | isopentenyl-diphosphate delta-isomerase | Hydrolase activity |
| G0RWQ8 | 7.68 | Ubiquinol-cytochrome C reductase-like protein | Mitochondrial respiratory chain complex III assembly |

Supplementary Table 3: Top SSDA upregulated in *B. velezensis* treated *L. fungicola*

| **Protein IDs** | **Actual difference** | **Description** | **Function** |
| --- | --- | --- | --- |
| G0RKC4 | 7.83 | FKBP-type peptidyl-prolyl cis-trans isomerase | peptidyl-prolyl cis-trans isomerase activity (stress response) |
| G0RMM8 | 7.77 | Isovaleryl-CoA dehydrogenase | acyl-CoA dehydrogenase activity |
| G0RJG3 | 7.13 | SPFH domain family protein | Plasma membrane component |
| G0RE21 | 6.86 | isocitrate lyase | carboxylic acid metabolic process |
| G0RDJ7 | 6.20 | glycosyltransferase family 66 protein | protein glycosylation |
| G0R8P2 | 5.94 | GTP-binding protein rho2 | GTPase activity, GTP binding |
| G0RBS0 | 5.35 | ubiquitin-activating enzyme E1 1 | Protein Ubiquitination |
| G0RQC8 | 4.33 | MMS19 nucleotide excision repair protein | Iron-sulfur cluster assembly, DNA repair |
| G0RKZ7 | 4.02 | Aldehyde dehydrogenase | Oxidoreductase activity |
| G0RJG5 | 3.85 | Vacuolar ATP synthase subunit D | Vacuolar transport, proton transmembrane transport |

Supplementary Table 4: Top SSDA downregulated in *B. velezensis* treated *L. fungicola*

| **Protein IDs** | **Actual difference** | **Description** | **Function** |
| --- | --- | --- | --- |
| G0RQS7 | 55.53 | manganese superoxide dismutase | Removal of superoxide radicals, superoxide dismutase activity, metal ion binding |
| G0RSJ8 | 45.04 | 60S ribosomal protein L5 | Translation |
| G0RTH1 | 32.97 | 40S ribosomal protein S30 | Translation |
| G0R734 | 32.05 | f-type h+-transporting atpase subunit h | ATP synthesis coupled proton transport |
| G0RTN4 | 29.12 | hydantoinase B/oxoprolinase | Hydrolase activity |
| G0RR73 | 28.98 | 60S ribosomal protein L12 | Translation |
| G0RLM5 | 16.59 | 3-isopropylmalate dehydrogenase | leucine biosynthetic process, branched-chain amino acid biosynthetic process |
| G0R900 | 14.33 | WSC domain-containing protein | cellular oxidant detoxification, cellular response to DNA damage stimulus |
| G0RWQ8 | 12.59 | ubiquinol-cytochrome C reductase-like protein | Aerobic respiration |
| G0RFB4 | 11.94 | NADP-dependent glycerol dehydrogenase | Carbohydrate metabolic process |
